# Mechanisms of intron gain and loss in *Cryptococcus*

**DOI:** 10.1186/gb-2008-9-1-r24

**Published:** 2008-01-30

**Authors:** Thomas J Sharpton, Daniel E Neafsey, James E Galagan, John W Taylor

**Affiliations:** 1 Department of Plant and Microbial Biology, University of California at Berkeley, Berkeley, CA 94720, USA; 2 Microbial Analysis Group Broad Institute of MIT and Harvard, Cambridge Center, Cambridge, MA 02142, USA

## Abstract

**Background:**

Genome comparisons across deep phylogenetic divergences have revealed that spliceosomal intron gain and loss are common evolutionary events. However, because of the deep divergences involved in these comparisons, little is understood about how these changes occur, particularly in the case of intron gain. To ascertain mechanisms of intron gain and loss, we compared five relatively closely related genomes from the yeast *Cryptococcus*.

**Results:**

We observe a predominance of intron loss over gain and identify a relatively slow intron loss rate in *Cryptococcus*. Some genes preferentially lose introns and a large proportion of intron losses occur in the middle of genes (so called internal intron loss). Finally, we identify a gene that displays a differential number of introns in a repetitive DNA region.

**Conclusion:**

Based the observed patterns of intron loss and gain, population resequencing and population genetic analysis, it appears that recombination causes the widely observed but poorly understood phenomenon of internal intron loss and that DNA repeat expansion can create new introns in a population.

## Abstract 2

Comparison of five relatively closely related yeast *Cryptococcus* genomes suggests that recombination causes internal intron loss and that DNA repeat expansion can create new introns in a population.

## Background

The evolutionary dynamics of spliceosomal introns has remained a subject of considerable debate since their discovery over 30 years ago [1]. Although the dynamic nature of gene structure is well documented - intron position is not always conserved between orthologs - the frequency of intron gain and loss and the mechanisms behind these processes remain controversial subjects. Indeed, single gene analyses have identified examples of both gain and loss, but such single case studies are limited in scope and, consequently, trends and patterns are difficult to identify. With comparative genomics, many orthologs can be evaluated simultaneously. Thus, enough events can be characterized to identify trends and to infer mechanisms pertinent to intron evolution. Several studies have employed such techniques across deep phylogenetic distances to evaluate the relative contributions of intron gain and loss [2-4]. While successful at demonstrating the plasticity of eukaryotic gene structure evolution, the long times for evolutionary events associated with comparisons of deep divergence can confound estimation of the true number of events and elucidation of mechanisms responsible for these events [5]. Comparative analysis of recently diverged taxa may improve estimation of the number of intron gains and losses and identify the mechanisms responsible for the evolution of this aspect of gene structure. In this regard, the *Cryptococcus* species complex offers the ideal system to study the mechanisms of intron gain and loss.

**Figure 1:**
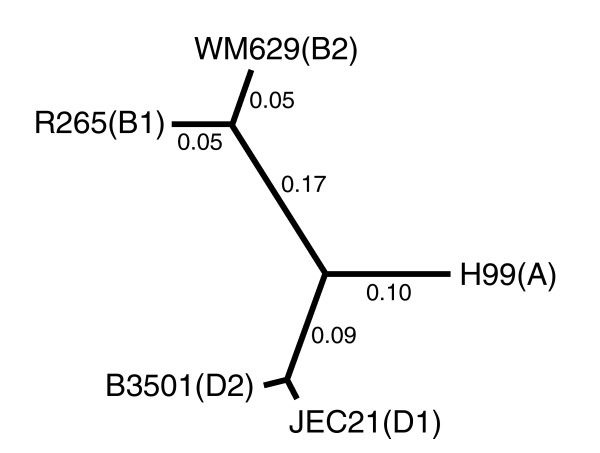
*Cryptococcus *genome sequence phylogenetic tree. A phylogeny of the five *Cryptococcus *strains used in this study. Phylogeny generated from neighbor-joining analysis of a concatenation of all five-way orthologs. Serotype identifiers are indicated in parentheses next to the corresponding strain names. Branch lengths indicate synonymous divergence (dS; not shown for values <0.01). JEC21 is the reference strain.

*Cryptococcus neoformans *and *Cryptococcus gattii *speciated less than 100 million years ago, so comparing these two species should avoid the problems of deep divergence while allowing enough time for intron gain and loss to occur [6,7]. A Basidiomycete yeast that causes the disease cryptococcosis in humans [8], *Cryptococcus *is subdivided into four serotypes. Currently, genome sequences are available for five strains spanning three of the serotypes (A, D1 and D2 from *C. neoformans *and B1 and B2 from *C. gatii*; *C. gatii* serotype C is not represented by a genome sequence; Figure 1). Additionally, D1 has particularly good gene structure annotation due to an extensive expressed sequence tag (EST) database (approximately 23,000 sequences) [9]. By evaluating the conservation of introns across the *Cryptococcus *clade, we estimated the rate of intron loss in *Cryptococcus*, identified genes that rapidly lose introns, propose an intron loss mechanism and discovered a gene that gained two introns through DNA repeat expansion.

## Results

### Estimation of divergence time

In a previous study, Xu *et al*. [7] used a four-gene phylogeny to estimate that *C. neoformans *and *C. gatii *diverged roughly 37 million years ago. We evaluated this prediction by estimating the silent substitution rate (dS) from all aligned orthologs from the five available *Cryptococcus *genomes. Pairwise comparisons between serotypes A/D and B identified dS estimates of approximately 0.32. Previous work found that the Eurotiomycete neutral mutation rate ranges from 1e-8 to 1e-9 mutations per year depending on the method of rate calculation [10]. Thus, assuming midpoint rooting and a molecular clock rate of evolution similar to the Eurotiomycetes, we estimate that *C. neoformans *and *C. gatii *diverged between 16 and 160 million years ago. Employing a commonly applied neutral mutation rate of 2 × 10^-9 ^suggests that the two species diverged 80 million years ago, doubling previous estimates that applied these same assumptions [7,11]. However, because of the difficulty of equating sequence divergence with time, we report all rate calculations and comparisons in terms of this date approximation as well as dS. For the purposes of identifying fixed structural changes by parsimony, we consider D1 and D2 to be the same species, and, therefore, consider the branch subtending these two strains to be a terminal branch. We calculate the total terminal branch length to be 0.29 substitutions per silent site, and use this figure to calculate the rate of observed gene structure changes on terminal branches.

### Intron loss predominates

The reference strain, D1, has an intron-dense genome with 36,275 introns across 6,954 genes (average of 5.5 introns per gene). *Cryptococcus *thus exhibits a density more similar to humans (7.8) than to other fungi, such as *Saccharomyces cerevisiae *(0.05), *Neurospora crassa *(1.77) or *Ustilago maydis *(0.75) (from [12]). This relative intron abundance may be driven by intron gain, a rarely observed phenomenon across such short phylogenetic distances. Thus, we sought to determine the relative contribution of intron gain and loss in the evolution of *Cryptococcus *gene structure.

**Figure 2:**
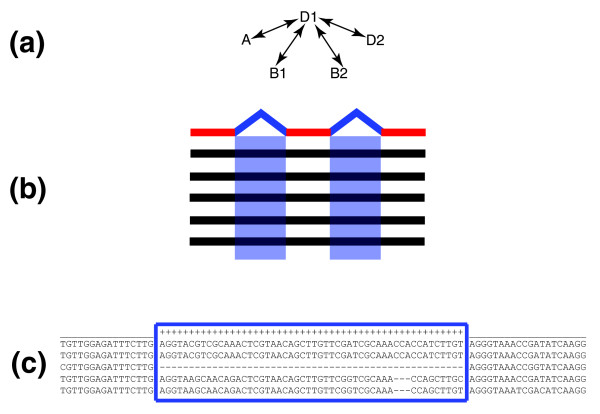
Comparative analysis method employed. A three-step process identified gene structure variation. **(a) **D1 orthologs were identified in the other strains' genome sequences via best bidirectional BLASTn and syntenic conservation. **(b) **Orthologous sequences were then aligned (black lines) and the D1 gene structure was overlayed (red lines are exons, blue boxes are introns). **(c) **Introns were scanned for cases of precise intron excision relative to the reference strain D1.

We aligned orthologs of D1 genes from four other *Cryptococcus *genomes and searched for D1 introns that were missing in at least one other taxon. We evaluated 33,473 D1 introns across 5,700 orholog sets and identified 49 cases where at least one ortholog was lacking an entire intron in an unambiguous alignment (Figure 2). Using parsimony analysis to distinguish intron gains from losses, it appears that intron loss significantly predominates over gain along the evolution of the *Cryptococcus *clade. Of the 49 events, there are 31 cases of unambiguous loss along a lineage. We found no instances of unambiguous gain. There remain 18 unresolved events due to an inability to infer the ancestral state of the character. A search for orthologous introns in *U. maydis*, *Phanerochaete chrysosporium *and *Coprinus cinereus *failed to resolve these changes as gain or loss. If each loss is considered an independent event and all 49 events are assumed to be losses, the fastest approximate intron loss rate of 1.04 × 10^-11^ introns per year (5.2 × 10^-3 ^times dS) can be inferred (1 - x^1/T^), where x is the proportion of retained ancestral introns and T is the time since divergence (estimated from terminal branch dS), as in [3]. Resampling analysis of the 49 intron deletion events provided a 95% confidence interval of this rate of 9.67 × 10^-12 ^to 3.08 × 10^-11 ^(4.84 × 10^-3 ^to 1.54 × 10^-2^). This intron loss rate is significantly slower than that previously calculated for *Schizosaccharomyces pombe *(2 × 10^-9^), and may, in part, account for the relatively high intron density in *Cryptococcus *[3].

### Some genes preferentially lose introns

Introns have been popular phylogenetic markers under the assumption that gains and losses are relatively rare and unlikely to be the result of parallel evolution [13]. However, analysis of the Dipteran *white *gene warned that some genes might be prone to parallel gene structure changes [14]. Additionally, studies have shown that some highly expressed genes are prone to losing introns and may be evolving more rapidly than previously assumed [15]. We examined *Cryptococcus *gene structure evolution to identify genes that rapidly undergo intron gain or loss. Because of the phylogenetic structure of the taxa involved in this study and the restriction of the analysis to serotype D introns, it is impossible to resolve true parallel loss of an intron in serotype A and in serotype B taxa (as it would appear to be a gain in the serotype D clade). Thus, we searched for loci that lost different introns along multiple branches. Assuming that the 49 gene structure changes are all losses, the probability that a gene will lose an intron is 1.5e-3. Assuming, then, that there are five introns in a gene, the probability of a single gene losing two independent introns is 1.13e-5. Because such events are unlikely to occur by chance alone, the identification of such events suggests that some genes preferentially lose introns. We identified two loci that appear to rapidly evolve their gene structure, losing single, different introns along independent lineages. *CNE00990*, a ubiquinone biosynthesis-related protein that ancestrally exhibited 5 introns, lost its first intron in B1 and its fifth intron in A. Similarly, *CNF02410*, a cytoplasmic protein containing five ancestral introns, lost its second intron in A and its fourth intron in B1. Both loci are single copy in the genome and are supported with ESTs. If we treat the adjacent intron losses observed as single loss events, these relatively rapid gene structure changes constitute 11.1-22.2% (depending on whether the ambiguous events are true losses) of the losses observed. Though the sample size is small, this suggests that a sizable number of gene structure changes in *Cryptococcus *may not be stochastic, and may help to explain why parsimony-based inferences of intron loss over larger evolutionary distances tend to underestimate parallel losses and, consequently, overestimate intron gains [16].

### Evaluation of a common intron loss mechanism

**Figure 3:**
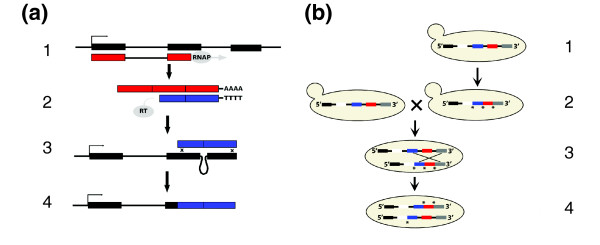
Poly-A primed mRNA derived intron loss. **(a) **Reverse transcriptase mediated intron loss. Step 1: a gene is transcribed by RNA polymerase (RNAP) and the transcript contains both exons (black boxes) and introns (lines between boxes). The transcript is processed to mature mRNA by splicing out introns and adding a poly-A tail to the 3' end. Step 2: reverse transcriptase (RT) primes off of the poly-A tail to create a cDNA (blue boxes), but falls off in a length-dependent fashion. Step 3: the partial cDNA then recombines with the gene. Step 4: the resulting gene has lost any introns that span the recombination junction. **(b) **Meiotic recombination can create internal intron losses. An individual from a population of *Cryptococcus *starts with a four-intron wild-type allele (step 1; exons are colored boxes). A subsequent poly-A primed mRNA-mediated mutation of said allele generates a 3' loss allele in this individual (as indicated by asterisks, introns 2, 3, and 4 are lost). This individual mates with another individual in the population that carries the wild-type allele (step 2). During meiosis, the alleles may pair and recombine, as in step 3. Depending upon the location of the recombination event (crossing lines in step 3), various recombinant progeny alleles will be generated (step 4). The final product is a 3' loss allele (missing introns 3 and 4) and an internal intron loss allele (missing only intron 2). Assuming neutrality, both recombinant alleles have an equal probability of eventually being fixed in the population.

The mechanism of intron loss has been the subject of considerable debate [17]. While several mechanisms may work in concert to create the observed loss patterns, the best-supported mechanism involves reverse transcription of a spliced mRNA and homologous recombination between the resulting intron-free cDNA and the intron containing genomic locus (Figure 3a) [18]. Additionally, to explain the relative paucity of introns at the 3' end of most genes in most eukaryotic genomes (Additional data file 2), it has been suggested that priming of reverse transcriptase occurs at the poly-A tail on the 3' end of the mRNA [19,20], which favors 3' intron loss over 5' loss via recombination of partial cDNAs. This mechanism should favor the loss of consecutive introns beginning from the 3' end and all losses should show precise intron deletion of sequence between the splice sites. To test the hypothesis of spliced mRNA intron loss, we evaluated the pattern of intron loss in *Cryptococcus*.

First, we investigated adjacent intron loss by searching for multiple, adjacent intron losses and then determined the likelihood that the losses were due to a single evolutionary event. Some genes have lost more than one intron, as evidenced by the observation that 31 parsimoniously explained intron losses occurred in only 16 loci. Specifically, genes *CNI01550*, *CNN02320*, and *CNA01350 *unambiguously demonstrate multiple, consecutive intron losses (Additional data file 1). Genes *CNK02730 *and *CNB02430 *also have consecutive introns missing in orthologs, but it is uncertain if these changes are the result of intron loss. In their study of fungal introns, Stajich and Dietrich [21] employed a comparative method that resolved the events in *CNK02730 *as losses. Here, given the relative rarity of intron gain and the lack of any clear mechanism that would enable multiple adjacent gains, we treat the changes in these two genes as losses. However, without the necessary phylogenetic support, characterization of these particular events is not conclusive.

Multiple adjacent intron loss may be caused by a single evolutionary event (that is, the spliced mRNA based model) or by multiple, independent losses. To distinguish between these hypotheses, we generated a loss distribution for each gene demonstrating adjacent loss via a resampling analysis under the assumption that losses were random and independent across a gene (see Materials and methods). These distributions were used to test the null hypothesis that the observed losses were the result of independent loss events within the gene (Additional data file 1). This analysis thus evaluates how intron loss is distributed across the length of a gene regardless of that gene's intron loss rate. In every gene except *CNA03150*, the null hypothesis could be rejected, supporting the hypothesis that one event accounted for all intron losses within a single gene. This observation of simultaneous adjacent loss suggests that an mRNA mediated loss mechanism exists in *Cryptococcus*.

**Figure 4:**
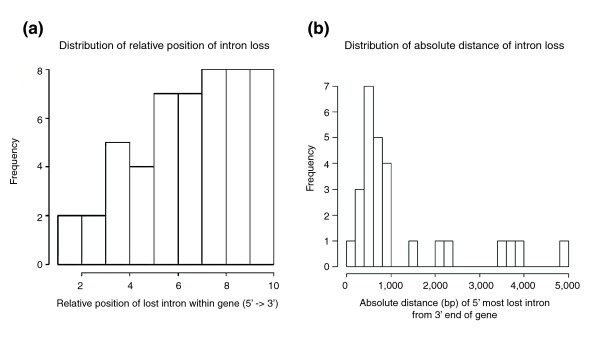
Distribution of lost introns in *Cryptococcus*. **(a) **Histogram of the location of an unambiguous intron loss event relative to the entire coding length of the gene. Loss events are binned according to the position (in base-pairs) of their occurrence in a coding sequence divided by the coding sequence length. **(b) **Histogram of absolute distance between a lost intron and the 3' end of its transcript. Since this is an evaluation of transcript based intron loss, adjacent intron losses are considered single events and the distance for such cases involves only the 5' most intron.

Given, then, that cDNA likely plays a role in intron loss, we evaluated whether these cDNAs originated at the 3' poly-A tail of the transcript. A histogram of the relative positions of lost introns in a gene indicates a 3' preference for intron loss (Figure 4a). We also investigated the relationship between intron loss and absolute distance from the 3' end of the gene. Most loss events occur within 1,000 bp of the poly-A tail (median = 672 bp; Figure 4b), but there are extreme cases where the enzyme would have had to synthesize over 4,000 bp of cDNA under this priming model. Additionally, most losses appear as internal islands in the gene; there are few lost introns that are followed by the loss of all downstream 3' intron positions. Only three events (including *CNK02730*) involve the loss of the 3' terminal intron (in the two other cases, it was the only intron lost) and there are not enough data to determine if 3' terminal losses are significantly more numerous than 5' terminal intron losses (one case). Furthermore, only one of the adjacent, multiple intron loss events previously described included a loss of the 3' terminal intron (*CNK02730*). Taken together, these findings suggest an alternative mechanism of loss or a variation on the simple poly-A primed mechanism previously described.

### Identification of a polymorphic, gained intron

We found no clear examples of completely new D1 introns; however, we did find a D1 gene with additional identical introns compared to A and D2. By submitting every intron to a BLASTn homology search against the rest of the genome, we identified the gene *CNN00420*, a single copy, six-intron gene in D1 in which introns 3, 4 and 5 are identical and differ from intron 2 by a single point mutation; intron 6 has additional 3' sequence. The introns in this gene are supported by unique ESTs, indicating that the gene is functional and that splicing occurs at the canonical splice sites (GT|AG). The orthology detection method employed in the previously discussed comparative analysis failed to detect this locus due to the lack of identifiable orthologs in the serotype B genomes. By relaxing this criterion, orthologs were identified in A (*CNAG_06312 *[12]) and D2 (GenBank: CNBN0400). The subsequent alignment identified two D1 intron sequences (introns 3 and 5) that correspond to gaps in the orthologs, implicating gains in the D1 lineage. Examination of *CNN00420 *reveals two types of repeats in the gene: short, internal exonic repeats (approximately 39 nucleotides in length) and longer repeats that include the ends of exons as well as a full length intron (approximately 107 nucleotides in length) (Additional data file 4). The alignment between the D1 and D2 orthologs indicates that the creation of extra copies of this longer repeat was responsible for the intron gains in the D1 sequence.

To rule out genome sequencing and contig assembly artifacts, we resequenced *CNN00420 *from the *Cryptococcus *serotype D population (strains TP0603, WM629, JEC20 (sib to D1), B3502 (sib to D1 and parent to D2) and genomic strains D2 and D1). Our new sequence verified that the genome sequences are correct and showed that the intron in D1 is shared with sib JEC20 and parent B3502 (Additional data file 5). Sequence from the additional individuals demonstrates a differential number of D1 intron containing repeats among the strains, suggesting that an expansion or contraction in the number of repeats accounts for the unique sequence in D1 (Additional data file 6). Because A lacks the introns and because the introns lay within a repeat, the most parsimonious explanation for the unique intron sequence is that it was gained by duplication in D1.

## Discussion

*Cryptococcus *genes are more intron-dense than those of other fungi [9], suggesting that *Cryptococcus *either gained introns at a faster rate or lost introns at a slower rate than other fungi. A screen of 33,473 introns across five taxa identified 31 intron losses and no intron gains. Because of a lack of phylogenetic resolution, an additional 18 gene structure changes could not be unequivocally attributed to intron gain or loss. While some of these unresolved events could be gains, a previous analysis of *Cryptococcus *gene structure evolution that screened for intron gain in the serotype A and B lineages also found no clear cases of gain [21]. If introns have been gained since *Cryptococcus *diverged, then they have done so at a much lower rate than losses or in such a way that makes detection difficult. Intron loss appears to be a rare event (0.09% of introns evaluated were lost) in *Cryptococcus*, occurring, under conditions assuming the most possible loss, at a rate of 1.04 × 10^-11^ introns per year (5.2 × 10^-3^ time dS), or 4.33 × 10^-12^ introns per year (2.17 × 10^-3^) if adjacent losses are single events. Conversely, the intron loss rate in *S. pombe *is 2 × 10^-9 ^introns per year [3]. It should be noted that the *S. pombe *rate, the only other intron loss rate calculated in fungi, was estimated by comparing *S. pombe *to the Opisthikont ancestor (estimated divergence approximately 1,500 million years ago). As the rate of intron loss may not be constant, there could have been moments during the much longer period assessed for *S. pombe *that matched the rate that we calculated over 80 million years of *Cryptococcus *evolution. These results suggest that the relatively large intron density of *Cryptococcus *compared to other fungi is not the result of a faster rate of intron gain, but rather a slower rate of intron loss.

Not all genes in the genome, however, appear to lose introns at the same rate. We identified two loci that have lost different introns along multiple, independent lineages. These gene structure changes are unlikely the result of chance and may be driven by some gene specific bias to lose introns. Whatever the cause, this finding suggests that introns may be lost in parallel over relatively recent time scales. This should raise caution about using an intron as a phylogenetic marker, a relatively common technique in evolutionary analysis. Further studies of such genes may help identify any selective pressures associated with relatively unstable gene structures.

We identified five genes that lost several adjacent introns, a pattern indicative of a spliced mRNA-intermediate intron loss mechanism (Figure 3a) [10]. If reverse transcriptase creates cDNA by initiating at the poly-A tail of the transcript and if homologous recombination between the ends of the spliced cDNA and the intron-containing gene causes intron loss, then we would expect to identify a bias towards 3' intron loss and a single loss event should frequently remove multiple introns and always include the 3' terminal intron in the loss. We do observe a bias towards 3' intron loss (Figure 3a), but when we evaluate where intron losses occur, they are most frequently internal; we rarely observe the loss of the 3' terminal intron. While it may be argued that natural selection may favor 3' intron retention, studies on this subject indicate otherwise, observing only a 5' intron retention bias [22]. It is noteworthy that *Cryptococcus *is not the only organism in which internal intron loss predominates. A previous analysis found that extraordinarily long exons, which result from intron loss, accumulate internally in the genes of many Eukaryotes [23] and a survey of Ascomycete gene structure evolution identified an internal intron loss bias [4]. Given the diversity of these observations, a general mechanism of internal intron loss should be considered.

Several mechanisms could explain internal intron loss via a spliced mRNA intermediate. First, the points of homologous recombination between the cDNA and the gene may occur upstream of the 3' most intron, resulting in its persistence by being excluded from the double cross-over event. Given that the mechanism proposed for intron loss involving mRNA is the same as that proposed for the origin of processed pseudogenes, if internal recombination was common, one would expect to find processed pseudogenes that are internal segments of genes. However, to our knowledge, there are few examples of processed pseudogenes that are internal gene segments [24]. Another point arguing against internal recombination is the model that the ends of cDNA molecules, and not internal regions, which necessarily exclude the 3' intron, promote cross-over through double strand break machinery [25]. In the same publication, Hu proposes a different model for internal intron loss. Here, intron loss could occur when an intron experiences a double strand break and if cDNA is used to repair the damage. However, this model would result only in single intron losses and could not explain our observations of adjacent intron loss.

Perhaps the mechanism with the best support is so-called self-primed reverse transcription. Here, internal initiation of reverse transcriptase occurs because the 3' end of the mRNA folds back onto the transcript and complementary pairs with some upstream region of mRNA (resulting in a hairpin in the secondary structure). cDNA is then synthesized from the region upstream of this base-pairing. This mechanism is suspected to cause the *4f-rnp *intron loss polymorphism in *Drosophila *[26] and is implicated in the loss of internal introns in many eukaryotes, including several fungi (*Aspergillus nidulans*, *Fusarium graminearum*, *Magnaporthe grisea*, *N. crassa*, *Plasmodium falciparum*, *S. pombe*, and *U. maydis*) [22]. While subsequent mutation impedes our ability to determine if this mechanism contributes to intron loss (for example, erosion of the complementary sequence), the pattern of loss observed in *Cryptococcus *suggests a different mechanism contributes to internal intron loss. Because there is no known bias regarding where, along the length of a transcript, the 3' end can complementary pair, this hairpin-based loss mechanism should produce intron losses along the length of a gene with equal probability. The majority of losses, however, cluster toward the 3' end of the gene (Figure 4a). Additionally, while there are losses that occur at extreme distances from the 3' end of the gene in *Cryptococcus *(Figure 4b), all losses are at distances smaller than the known, active fungal retrotransposons. Reverse transcriptase is, then, capable of synthesizing cDNA from the transcript's poly-A tail to these distant positions. Indeed, the only observation that does not fit the requirements of the poly-A primed mechanism is that intron loss events frequently exclude 3' terminal introns. Given this loss pattern, we propose a simple explanation to account for the observation of internal intron loss (shown in Figure 3b). If, within a population, an allele that had lost a series of introns via recombination with a poly-A initiated cDNA were to undergo meiotic recombination with an allele that retained 3' introns, the pattern of internal intron loss could be created.

To determine if this mechanism is likely, we employed an allelic sojourn time density function derived from a diffusion approximation to estimate the frequency with which an initially single-copy, 3' poly-A primed intron loss allele recombines with a wild-type allele before being absorbed or fixed in the population (see Materials and methods). While we cannot be certain about historical recombination rates, we can make a reasonable estimation by employing the measured meiotic recombination rate determined for *C. neoformans *serotype D [27]. Our analysis estimates that 'internal intron loss' alleles are generated at a rate 2.08 times that of '3'-generated intron loss' alleles. Assuming equal probability of fixation, we therefore might expect approximately twice as many genes to exhibit internal intron loss as genes exhibiting a 3' loss of two or more introns, even though all intron losses are originally caused by reverse transcription from the 3' poly-A tail. This model for internal intron loss is built upon an experimentally verified method of cDNA creation (initiation at the poly-A tail), explains the relative clustering of 3' intron loss and, assuming meiotic recombination occurs at random positions between the cDNA and the gene, accounts for the diverse distribution of losses observed (Additional data file 3). To our knowledge, this is the first time such a mechanism has been described to explain the observation of internal intron loss.

While we found no intron gains between the two *Cryptococcus *species, we did identify a gene that has gained introns within *C. neoformans*. The D1 gene *CNN00420 *contains unique intron sequences relative to orthologs in A and D2 (no orthologs could be identified in serotype B). Resequencing strains from the serotype D population confirmed that these introns in the D1 allele are unique, implicating a sequence gain. Because each gained intron has exact identity to three other introns in the gene (and near exact identity to two others), it is likely that unequal crossing over, gene conversion or repeat expansion through replication strand slippage caused the gains. While we cannot discern which of these DNA duplicating mechanisms caused the intron gains in question, several studies have shown that replication strand slippage is a common and frequent means of varying intergenic repeat copy number. In *Escherichia coli*, RecA independent duplications and deletions between repeats of several hundred bases have been shown to occur at high frequency (10^-5 ^to 10^-4 ^per cell generation) [28]. In an elegant analysis of FLO gene repeat variation in yeast, Verstrepen *et al*. [29] concluded that replication slippage is the cause of the observed length polymorphisms and that the repeats could be at least 100 nucleotides in length. Here, we propose its role in generating new introns in *CNN00420 *(Additional data file 7).

To our knowledge, there is only one other example of an intron gain occurring through repeat duplication. Knowles and McLysaght [30] suggested that this mechanism is responsible for a gained intron in the *TOUCH3 *gene relative to its paralog in *Arabidopsis*. However, though there is debate on this point [31], paralogs may undergo wholly different gene structure evolution than orthologs [2]. Additionally, the role of the *Arabidopsis *whole genome duplication on gene structure evolution is uncertain. Our analysis verifies that this mechanism can create introns in orthologs and contributes to gene structure divergence between species. Furthermore, this is the first time a DNA repeat expansion has been observed to create an intron polymorphism.

In his study of intron gain rates, Roy noted that certain ancient lineages underwent big bursts of intron gain while subsequent lineages experienced a precipitous decline in intron gain rates [3]. This suggests that these early lineage introns may have been created via a wholly different mechanism than those created in contemporary times. While we do not suspect that intron containing repeat expansion is the mechanism responsible for the origin of most introns, it appears to be a more widespread mechanism than previously expected and may be an important contemporary means of creating new intronic sequence in the genome. As additional closely related genome sequences become available, future comparative genomic studies should verify whether this mechanism of gain is ubiquitous and identify other contemporary mechanisms that create introns.

## Materials and methods

### Genome sequences utilized

The *C. neoformans *serotype D strain JEC21 (D1) genome sequence and annotation was obtained from GenBank as deposited by TIGR (January 2005, 20 Mb, 14 chromosomes). A second serotype D strain sequence, B3501 (D2), was obtained from the Stanford Genome Technology Center (June 2004 assembly, 18.5 Mb, 70 contigs). The *C. neoformans *serotype A strain H99 (A) was sequenced by the Broad Institute (October 2004 assembly, 19.5 Mb, 210 contigs). The Broad Institute also sequenced the *C. gatii *serotype B strain R265 (B1) (January 2005 assembly, 17.2 Mb, 28 contigs). The second serotype B strain WM276 (B2) was obtained from the BC Genome sequencing center (assembly dated March 2004, 18.0 Mb, 33 contigs).

### Comparative analysis

Whole genome alignments were created through a multi-step process using D1 as a reference. First, pairwise alignments between D1 and the other sequenced strains were created using PatternHunter [32]. Blocks of four-way homologous contigs were then identified using a hierarchical synteny-clustering algorithm as described in [33]. Multiple alignments of homologous regions were generated using Multi-LAGAN [34].

As described in [6], the annotation for D1 is well supported because an extensive EST library was used to train the gene structure predictive algorithms. Thus, we aligned orthologs to each D1 reference gene and evaluated the conservation of each annotated D1 intron across the alignment (Figure 2) [35]. We identified orthologs of D1 genes by searching for best all-way reciprocal BLASTn hits against the corresponding genomes that also demonstrated syntenic conservation. Because previous studies have demonstrated differential tempos and modes of intron evolution between paralogs [30], we elected to use this type of conservative ortholog detection method [30]. To enable comparative analysis, each orthology cluster had to include a sequence from serotype B, which represented the outgroup of the comparison.

Clusters of orthologs meeting these criteria were aligned separately from the whole genome alignment using the mLAGAN multiple sequence alignment algorithm and the phylogeny shown in Figure 1 as the guide tree. To identify introns in these alignments, Perl scripts were constructed to overlay the D1 gene annotation onto the alignment. We subsequently identified sequences with gaps that completely spanned an intron position. When we detected missing introns, the quality of the gene alignment was evaluated by eye and the principles of parsimony were used to infer whether the intron in question had been gained or lost. For example, if A is missing an intron sequence that is found in D1, D2, B1 and B2, then we score the sequence as an intron loss in A. It should be noted that parsimony has been shown to overestimate the relative contribution of intron gain at large phylogenetic divergences by effectively undercounting the number of parallel loss events [16]. The close evolutionary distance involved in this study should mitigate this problem.

The unrooted phylogenetic topology among the five sequenced strains was determined by a neighbor-joining analysis [36] on a concatenation of all orthologous open reading frames. Branch lengths based on silent site divergence observed across a concatenation of 5,700 aligned ortholog clusters were estimated using the codeml application (model = 0, NSsites = 0, ncatG = 1) from the PAML 3.15 package [37].

### Resequencing analysis

Genome sequenced strains JEC21(D1) and B3051(D2), as well as additional serotype D individuals TP0603, WM629, JEC20 and B3502, were generously provided by W Meyer. The method of Bolano was used to extract genomic DNA from strains grown in liquid culture [38]. Conserved regions of the alignment between the D1 and D2 genome sequences were used to construct PCR primers for touchdown PCR. Amplified target DNA was cloned with Invitrogen's TopoTA cloning kit (Invitrogen, Carlsbad, CA, USA) and subject to standard BigDye (Applied Biosystems, Foster City, CA, USA) sequencing procedures as in [39]. Sequencher (v4.2.2, Gene Codes Corp., Ann Arbor, MI, USA) was used to identify high quality sequence reads and BioEdit's CAP contig assembly tool was used to construct full length sequences [40]. Initial alignments were generated by ClustalW and edited by hand [41].

### Diffusion approximation

We applied a sojourn time density function derived from a diffusion approximation to estimate the probability that a recombination event between a 3' generated intron loss allele (defined in Results) and a wild-type allele could result in an allele missing only internal introns (Additional data file 8) [42-44]. Let *y *represent the vector {x, N, *s*, *h*}, where x, 0 ≤ x ≤ 1, represents the frequency of an element in a population of N individuals, *s *is the intensity of selection acting on the allele and *h *is a heterozygous fitness factor. A newly created element thus appears in the population at a frequency of 1/2N. Under standard assumptions of the diffusion approximation, the amount of time, in N generations, an initially single copy element spends in the population prior to being absorbed (x = 0) or fixed (x = 1) is:

$$\tau [y]=\frac{2}{\left(v[x]\Psi [y]g[0,1]\right)}(g[1/2N,1]g[0,x]\theta [p-x]+g[0,1/2N]g[x,1]\theta [x-p])$$

where:

$$\begin{array}{c} m[y]=2Ns(1-x)x(h+x-2hx)\\ v[x]=x(1-x)\\ \Psi [y]=e^{-2\int \frac{m[y]}{v[x]}dx}\\ g[a,b]=\int_{a}^{b}\Psi [y]dx\\ \theta [z]=\{\begin{array}{l} 1,z>0\\ 1/2,z=0\\ 0,z<0 \end{array} \end{array}$$

as in [43]. Here, we assume that there is no selective pressure acting on intron retention (*s *= 0), that the population is sufficiently large (2N = 10e5), and that the only value for any x < 1/2N is x = 0. Because *s *= 0, we need not estimate *h*, the dominance coefficient. Under these assumptions, the frequency spectrum of alleles is given approximately by:

$$F[y]=\frac{\tau [y]}{\int_{0}^{1}\tau [y]dx}$$

Of interest is the frequency that an allele missing 3' introns recombines with a wild-type allele (no missing introns) to create a recombinant allele that is missing only internal introns. This can be estimated by defining the probability of recombination between dissimilar alleles as a function of allele frequency and integrating over the frequency spectrum as follows:

$$G[y]=\int_{0}^{1}\frac{\tau [y]}{\int_{0}^{1}\tau [y]dx}2NLrx(1-x)dx$$

Here, *r *is the recombination rate and *L *is the average exon length. In *Cryptococcus*, these values are 7.58e-7 recombination events per base-pair per generation [27] and 253 bp, respectively. Incorporating these values returns an expectation of 2.08 recombination events that would create 'internal intron loss' alleles for every '3 intron loss' allele generated by mutation, suggesting that, on average, 'internal intron loss' alleles are generated at a rate approximately 2.08 times that of '3' intron loss' alleles.

### Statistics

Resampling analysis was conducted to determine intron loss rate 95% confidence intervals. A total population of 33,473 introns (equal to the total number of screened orthologous introns) was established, where 49 introns in the population corresponded to loss events and the remainder were intron conservation events. We sampled, with replacement, 10,000 introns from this population and then calculated the frequency and subsequent rate. Calculations were conducted 10,000 times and a normal curve approximation of the subsequent rate distribution was used to calculate the 95% confidence interval.

Resampling analysis was also conducted for each gene demonstrating adjacent intron loss to determine the probability that the adjacent losses were caused by random, independent events. For each gene, a population equal to the number of introns in the gene was established, where each individual in the population has a value corresponding to an intron position within the gene. Samples equal to the number of observed losses in the gene were taken from this population, without replacement, and the longest consecutive run of adjacent introns was counted. After 10,000 runs, a histogram of the longest runs was created and used to evaluate the null hypothesis that the observed data were due to random, independent loss events.

## Abbreviations

A, *C. neoformans *serotype A strain H99; B1, *C. gattii *serotype B strain R265; B2, *C. gattii *serotype B strain WM276; D1, *C. neoformans *serotype D strain JEC21; D2, *C. neoformans *serotype D strain B3501; dS, silent substitution rate; EST, expressed sequence tag.

## Authors' contributions

TJS wrote and ran necessary software, performed computational and wet lab analyses, created the figures and tables and wrote the paper. DEN generated syntenic alignments for ortholog detection, generated the species phylogeny and contributed to the content and writing of the paper. JEG conceived of the computational project and provided access to appropriate resources. JWT conceived of the wet lab experiment and provided necessary resources, contributed to the content and writing of the paper and supervised TJS.

## Additional data files

The following additional data are available. Additional data file 1 is a table documenting the consecutive intron loss events. Additional data file 2 is a histogram of the relative distribution of introns within genes in the D1 genome. Additional data file 3 is a histogram of the order number of a lost intron, from 5' to 3' along the length of the gene, divided by the total number of introns in the gene. Additional data file 4 is a dot plot of *CNN00420 *against itself. Additional data file 5 provides the multiple sequence alignment (FASTA format) of *CNN00420 *orthologs from the serotype D population. Additional data file 6 shows *CNN00420 *gained introns along the D1 lineage. Additional data file 7 shows the mechanism of intron gain via strand slippage. Additional data file 8 provides our Mathematica source code for the sojourn time density approximation.

## Supplementary Material

Additional data file 1 Consecutive intron loss events. Click here for file

Additional data file 2 Introns are binned according to the position (in base-pairs) of their occurrence in a coding sequence divided by the coding sequence length. Click here for file

Additional data file 3 Intron loss in regard to intron order within the gene is well distributed, not requiring the 3'-most intron to be lost. Click here for file

Additional data file 4 The large number of off-center, parallel lines illustrates this gene's highly repetitive sequence. Click here for file

Additional data file 5 The strain relationships are designated in Additional data file 6. Positions with a sequence of 'X' designate the boundaries of D1 annotated introns. Click here for file

Additional data file 6 The strain phylogeny is determined based on the history of strain synthesis as described in Results. The corresponding aligned *CNN00420 *ortholog for each strain is graphically represented in the cartoon next to each strain identifer. Horizontal bars correspond to sequence alignment positions: red lines indicate nucleotide sequence while white gaps indicate indels. Vertical blue bars correspond to annotated D1 introns. The phylogeny and the presence of unique sequence in the alignment corresponding to introns 3 and 5 in D1, its sibling JEC20, and parent B3502, suggest the gain of introns 3 and 5 in D1. Click here for file

Additional data file 7 Exons (black lines) and introns (gold lines) are synthesized in the daughter strand during DNA replication. Repetitive sequences (red boxes) may create a looping out of the daughter strand and subsequent re-synthesis of the looped out region (here, an intron). After meiosis, the result is an allele with an extra repeat copy and, subsequently, an extra (acquired) intron. Click here for file

Additional data file 8 Mathematica source code for the sojourn time density approximation. Click here for file
