# Supplementary material for: Mechanisms of intron gain and loss in Cryptococcus
Source: Genome Biol. 2008 Jan 30;9(1):R24. doi: 10.1186/gb-2008-9-1-r24 (PMC2395259; doi:10.1186/gb-2008-9-1-r24)
Supplement: Supplementary file 1 [file gb-2008-9-1-r24-S1.doc]

Locus	Total Introns	# Missing	Lost Event	External Lo	Independent Losses?
CNI01550	21	10	Yes	No	p = 0
CNN02320	12	4	Yes	No	p = 0
CNA01350	14	2	Yes	No	p = 0.012
CNK02730	9	5	N/A	Yes	p = 0.4979
CNB02430	19	7	N/A	No	p = 0
